# Clinical Implications of a Missing Hepatic Segment of the Inferior Vena Cava: A Case Report

**DOI:** 10.7759/cureus.62857

**Published:** 2024-06-21

**Authors:** Carlos F Castillo Reina, Marcelo Valdés Hernández, Claudia J Rodriguez Reus, Adrián A Negreros-Osuna

**Affiliations:** 1 Radiology Department, Hospital Regional Institute of Security and Social Services for State Workers (ISSSTE) Monterrey, Autonomous University of Nuevo León, Monterrey, MEX

**Keywords:** absence of the hepatic segment of the vena cava, dextrocardia, cholangiocarcinoma (cca), polysplenia, situs inversus with dextrocardia, inferior vena cava anamoly

## Abstract

Anomalies of the inferior vena cava (IVC) are often discovered incidentally, yet they pose significant challenges in both diagnosis and treatment. This study discusses a case of a 50-year-old female with situs inversus and well-differentiated cholangiocarcinoma, emphasizing the rarity and complexity involved. Imaging studies confirmed the presence of dextrocardia, abdominal situs inversus, and an interrupted IVC with continuation to the azygos vein. A thorough understanding of IVC embryology is crucial, as it helps explain the pathogenesis of such anomalies, which arise from disturbances in the embryonic venous system leading to various anatomical variations. Managing an interrupted IVC is critical due to the potential for vascular complications, highlighting the need for preoperative awareness. This study underlines the importance of recognizing and properly managing these rare vascular anomalies to improve patient care.

## Introduction

Anomalies in the inferior vena cava (IVC) development are rare. For example, the absence of the hepatic segment of the vena cava has an incidence of 0.6% in the population [1]. Typically, these anomalies are associated with congenital heart disease and heterotaxy syndrome or polysplenia. The clinical presentation of these patients can vary widely, from being asymptomatic to presenting varicose veins in the lower extremities, stasis, and recurrent deep vein thrombosis since adolescence [2]. Understanding these malformations requires understanding the embryogenesis of the inferior vena cava.

This study presents a case of interruption in the hepatic segment of the IVC with continuity at the level of the azygos vein in a patient with metastatic cholangiocarcinoma. In this study, we will discuss the tomographic characteristics and clinical significance of this condition.

## Case presentation

A 50-year-old female with situs inversus presented with a three-month history of jaundice. An MRI conducted at another facility revealed a tumor in the common bile duct (CBD), leading to its dilation to 19 mm. As a result, she underwent a Whipple procedure.

The histopathological analysis revealed well-differentiated cholangiocarcinoma with pancreatic invasion, but there was no evidence of angiolymphatic or perineural invasion. However, three peripancreatic lymph nodes showed cholangiocarcinoma metastasis, including capsular rupture and spread into the surrounding adipose tissue. The patient was directed to oncology and treated with adjuvant chemotherapy, specifically cisplatin and gemcitabine.

**Figure 1:**
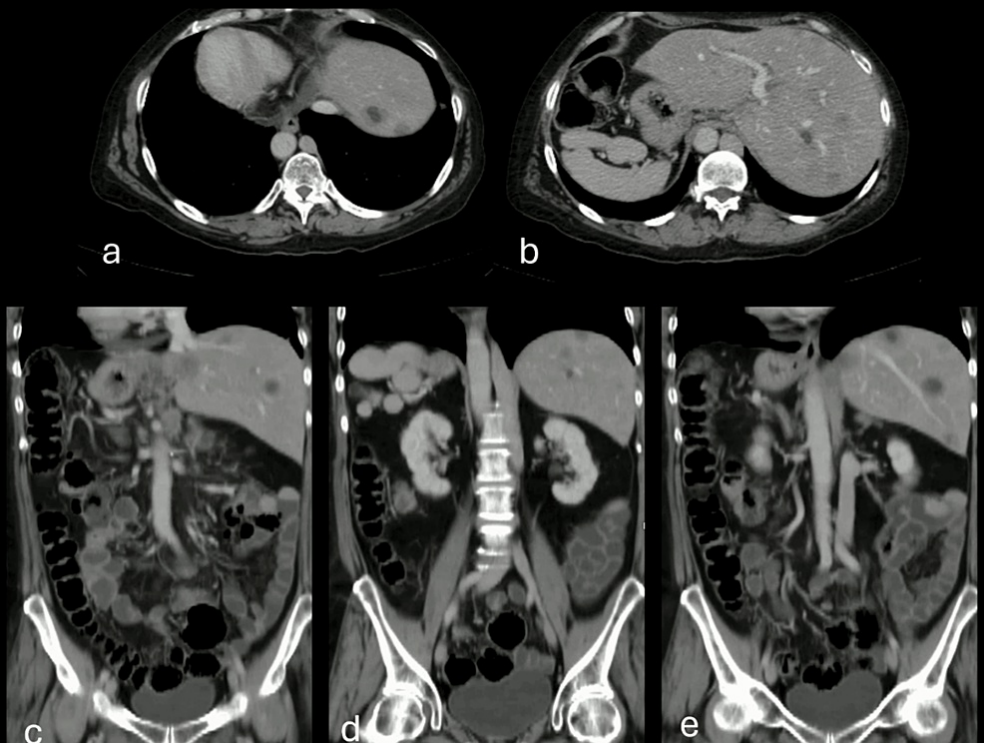
Detailed CT images of the patient. (a) The axial CT image shows the heart on the right side, indicating dextrocardia. (b) The axial image shows the liver on the left and the spleen, with accessory spleens on the right. (c) The coronal reconstruction image shows multiple well-defined liver lesions with peripheral enhancement and necrotic centers, post-Whipple surgery changes, and an absent hepatic IVC segment continuing into the azygos system and dextrocardia. (d) The coronal reconstruction shows polysplenia and abdominal situs inversus. (e) The reconstruction shows the colon on the right and the jejunum and ileum on the left, indicating intestinal malrotation. IVC: inferior vena cava

Consequently, she was referred for imaging to assess her response to treatment. At our facility, we performed a contrast-enhanced computed tomography (CT), revealing diffuse hepatic nodules indicative of metastases from the primary tumor. Additionally, the scan showed the absence of the hepatic segment of the inferior vena cava. However, the inferior vena cava continued upward behind the diaphragmatic pillars towards the azygos system, alongside polysplenia (Figures 1a-1e).

During her hospital stay, we performed a Doppler ultrasound, revealing partial chronic venous thrombosis in the common femoral and proximal femoral veins, with no involvement of the iliac-cavus axis. After that, she remained asymptomatic and was under the care of the oncology service, where she was receiving second-line palliative chemotherapy.

## Discussion

Conditions affecting the inferior vena cava (IVC) arise from abnormalities in embryonic development. Often, these are discovered incidentally and do not impact blood flow significantly. However, they may be associated with other vascular malformations. The prevalence of such conditions is between 0.2% and 0.5% in the general population and rises to 2% among individuals with other cardiovascular anomalies [3].

In this case, we described the absence of the hepatic segment of the inferior vena cava (IVC), where it continues as the azygos vein and has a prevalence of 0.6% [4]. A study by Fulcher and Turner reviewed 19 adult cases with situs anomalies, finding that seven cases involved polysplenia and seven cases featured an interrupted IVC with continuation into the azygos or hemiazygos vein [5].

The development of the inferior vena cava (IVC) occurs between the sixth and eighth weeks of embryonic development through the anastomosis of three pairs of primitive veins as follows: the posterior cardinal veins, subcardinal veins, and supracardinal veins. This process includes the involution of some of these anastomoses.

The absence of the hepatic segment of the IVC, continuing into the azygos vein, indicates a failure to form the right subcardinal-hepatic anastomosis, resulting in atrophy of the right subcardinal vein. Blood is then redirected through the retrocrural azygos vein, which partially derives from the thoracic segment of the right supracardinal vein [3].

Anatomical variants of the inferior vena cava (IVC) are primarily due to abnormal regression or persistence of embryological veins. While most anomalies are asymptomatic and found incidentally, they can lead to complications, such as lower extremity venous insufficiency, deep vein thrombosis, and pelvic congestion syndrome, and can affect the planning of vascular procedures. Common anomalies include duplication of the IVC, a left-sided IVC, and interruption of the IVC [6].

An interruption of the IVC with azygos continuation typically involves the interruption of the adrenal/intrahepatic segment. This condition is variously described as interruption, absence, abnormality, or agenesis [7]. In this scenario, the adrenal segment of the IVC diverts blood to drain through the azygos vein, while the hepatic segment only receives blood from the hepatic veins. It has a prevalence of 0.6% and is often associated with polysplenia, cardiovascular malformations, and situs anomalies [3].

Similar to cases where the infrarenal IVC is lacking, a disrupted IVC without adequate collateral pathways can lead to vascular complications like deep vein thrombosis and venous insufficiency. An enlarged azygos vein might be misdiagnosed as retrocrural lymphadenopathy or a right paratracheal mass, and a distended hemiazygos vein could be mistaken for a left-sided mediastinal mass [6]. Additionally, prominent collateral vessels could be confused with paraspinal masses. Prior recognition of these anomalies is essential for surgical planning, particularly before thoracic and cardiopulmonary bypass procedures.

Koc and Oguzkurt advocates for a simple nomenclature that categorizes all cases with absent or interrupted segments as "interrupted IVC," specifying the level of interruption and associated collateral pathways, such as "interrupted IVC (adrenal level) with azygos continuation" [7].

## Conclusions

Our case underscores the complex nature of inferior vena cava (IVC) anomalies, particularly when combined with conditions like situs inversus and cholangiocarcinoma, as seen in this patient. The rarity and variability of these anomalies highlight the need for comprehensive diagnostic evaluations and a multidisciplinary approach to treatment. A deep understanding of the embryological origins of IVC development is crucial for grasping the pathophysiology of these conditions and for informed clinical decision-making. Anticipated recognition of an interrupted IVC is critical to prevent vascular complications during surgical procedures. Further research is necessary to develop optimal management strategies, enhance patient outcomes, and refine treatments for those with complex vascular anomalies.
